# Effectiveness and safety of Chinese medicine combined with omeprazole in the treatment of gastric ulcer: A protocol for systematic review and meta-analysis

**DOI:** 10.1097/MD.0000000000025744

**Published:** 2021-04-30

**Authors:** Cheng Xie, Langhui Liu, Suyou Zhu, Mingquan Wei

**Affiliations:** a Huaibei Traditional Chinese Medicine Hospital; b Jiangxi University of Traditional Chinese Medicine; c Affiliated Hospital of Jiangxi University of Traditional Chinese Medicine, China.

**Keywords:** Chinese medicine, gastric ulcer, protocol, systematic review

## Abstract

**Background:**

Gastric ulcer (GU) is a common digestive system disease, and the main clinical manifestations are nausea and epigastric pain. In recent years, due to increased life pressure, unhealthy eating habits and environment, the incidence of gastric ulcer has increased year by year. Because the disease has a long treatment cycle and is prone to relapse, if it cannot be controlled in time, it can cause the disease to prolong, affect the daily life and health of the patient, and even cause complications such as upper gastrointestinal bleeding, ulcer perforation, and pyloric obstruction. Helicobacter pylori infection is one of the main causes of GU. Clinically, the curative effect of western medicine or traditional Chinese medicine cannot reach the ideal level, so in recent years, the combination of traditional Chinese and western medicine has been highly praised. The aim of this systematic review is to evaluate the effectiveness and safety of Chinese medicine combined with omeprazole for GU.

**Methods:**

The data and information will be retrieved from the databases of PubMed, Embase, Cochrane Library, CNKI, VIP, and Wanfang data. Literature search is limited to Chinese and English. The search time range is from the establishment of the database to April 7, 2021. The search strategy uses a combination of subject terms and free words to search. In order to avoid omissions, the search scope includes subject terms, keywords, or full text. Two reviewers will independently exclude substandard articles and extract eligible data. The risk of bias will be assessed using the Cochrane Handbook 5.1.0 for Systematic Reviews of Interventions. RevMan 5.3 will be used for systematic review and meta-analysis. This protocol will be reported according to the Preferred Reporting Items for Systematic Reviews and Meta-Analyses Protocols (PRISMA-P) statement, and the systematic review will be reported with the PRISMA statement.

**Results and conclusion:**

The efficacy and safety of Chinese medicine combined with omeprazole for the treatment of GU will be evaluated, and the conclusion will be published to provide medical evidence for a better clinical decision of patients with GU.

## 1 Introduction

Gastric ulcer (GU) is a chronic digestive system disease with a high clinical incidence and recurrence rate. The clinical manifestations are upper abdominal pain, abdominal distension, belching, and acid reflux. In severe cases, it may be accompanied by hematemesis and melena, or even may be complications such as gastric perforation, gastric bleeding, canceration, etc, have a serious impact on the patient's health and quality of life.^[1,2]^ Some scholars pointed out in the research that in patients with gastric ulcer, the positive rate of HP (Helicobacter Pylori) is 15% for those whose ulcer is located in the stomach. The positive rates of HP in patients with ulcers located in the antrum and horn of the stomach were 37% and 44%, respectively; males and females had significant differences in the positive rates of HP at each ulcer site, with the former having a higher positive rate.^[3]^ Western medicine clinically mainly uses triple therapy (proton pump inhibitor plus 2 antibiotics) to treat this disease. The treatment principle is to protect the gastric mucosa and inhibit the secretion of gastric acid. In the clinical treatment of GUs, acid suppression is the main measure. The commonly used drug omeprazole is a proton pump inhibitor, but in clinical practice, it has been found that the effect of omeprazole alone is not very satisfactory.^[4,5]^ Omeprazole can effectively reduce the secretion of gastric acid, reduce the digestion of gastric acid on the mucous membrane, and achieve the purpose of alleviating the symptoms of GU.

With the continuous development of traditional Chinese medicine in my country, in order to further improve the therapeutic effect of HP-related GUs, the combination of Chinese and Western medicine has become a new research direction. GU is classified as epigastric pain in traditional Chinese medicine in my country. It is caused by the weakness of the spleen and stomach, poor qi, and gastric disorders caused by eating disorder, fatigue, internal injury, and invasion of external pathogens. In addition, there is a certain relationship with emotional disorders. Ling^[6]^ compared the clinical efficacy of traditional Chinese medicine and omeprazole in the treatment of GU and the recurrence after 6 months of follow-up, she found that the effective rate of the traditional Chinese medicine group was higher than that of the omeprazole group and there were no cases of recurrence. Zhou et al^[7]^ compared the clinical efficacy and hemostasis time between standard triple therapy and additional traditional Chinese medicine in the treatment of GU. It is found that standard triple therapy plus traditional Chinese medicine is superior to triple therapy alone in terms of effective rate and hemostasis time.

At present, the treatment of GUs with weak spleen and stomach syndrome is often treated with acid-suppressing western medicines, soothing liver qi, and invigorating the spleen and stomach. However, the simple use of traditional Chinese and western medicines has certain disadvantages. Western medicines have a fast relief of clinical symptoms, but they have a higher recurrence rate. Chinese medicine has lower recurrence rate, but it has the disadvantage of slow relief of clinical symptoms. In order to further clarify its clinical application value, this study adopts the methods of systematic review and meta-analysis to comprehensively evaluate the effectiveness and safety of traditional Chinese medicine combined with omeprazole in the treatment of GU, and provide evidence-based basis for its rational use in clinical practice.

## 2 Protocol registration

The protocol of the systematic review has been registered in the INPLASY website (registration number is INPLASY202140048). https://inplasy.com/inplasy-2021-4-0048/. This systematic review protocol will be reported strictly adherence to the Preferred Reporting Items for Systematic Review and Meta-analysis Protocols (PRISMA-P).^[8]^

## 3 Materials and methods

### 3.1 Literature source

#### 3.1.1 Inclusion criteria

(1) Type of study: Only randomized controlled trials (RCTs) will be included, regardless of whether they are blinded or not, are limited to Chinese and English.
(2) Study participants: Patients who are clinically diagnosed with gastric ulcer through digestive endoscopy are not limited by gender, age, and course of disease. Participants with serious underlying diseases will be excluded.
(3) Types of interventions and comparators: The experimental group was treated with Chinese medicine decoction combined with omeprazole, and the control group was treated with omeprazole.
(4) Outcome indicators:
  1. Effective rate: Effective rate (%) = (number of cured cases + number of markedly effective cases + number of effective cases)/total number of cases × 100%.
  2. Incidence rate of adverse reactions.
  3. Recurrence rate of gastric ulcer bleeding.
  4. Time required for clinical symptom improvement.
  5. Ulcer surface healing.
  According to the results of gastroscopy, the clinical efficacy of patients was evaluated.^[1]^ The cure is that the gastroscopy shows that the ulcer has completely disappeared and the gastric mucosa has no inflammation; the obvious effect is that the gastroscopy shows that the area of the ulcer is reduced by >70%, and the inflammation of the gastric mucosa is significantly reduced; the effective is that the gastroscopy shows the area of the ulcer is reduced by 10% to 70%, and the inflammation of the gastric mucosa is reduced. Ineffective, the gastroscopy shows that the area of the ulcer is reduced or increased by less than 10%, and the inflammation of the gastric mucosa is not reduced, and the disease is even worse.

#### 3.1.2 Exclusion criteria

(1) Animal studies, reviews, pharmacokinetics, pharmacodynamics studies, etc.
(2) The trial design is not rigorous or the use of statistical methods is inappropriate.
(3) The results are selectively reported.
(4) Repeated literature.
(5) In addition to conventional treatment, add those who use other treatment methods to influence the judgment of the results.
(6) Those who do not mention clear clinical efficacy evaluation standards.

### 3.2 Data sources and search strategies

Computer search of PubMed, Embase, Cochrane Library, CNKI, VIP, and Wanfang data. Literature search is limited to Chinese and English. The search time range is from the establishment of the database to April 7, 2021. The search strategy uses a combination of subject terms and free words to search. In order to avoid omissions, the search scope includes subject terms, keywords, or full text. Search terms include: “traditional Chinese medicine,” “TCM,” “Chinese herbal medicine,” “Herbal medicine,” “Omeprazole,” “Proton Pump Inhibitor,” “PPI,” “Western medicine,” “Gastric Ulcer,” “GU,” “Peptic Ulcer,” “Randomized controlled trial,” “RCT.” The established search strategy for PubMed was displayed as follows:

Mesh term #1: ((traditional Chinese medicine) OR (TCM) OR (Chinese herbal medicine) OR (Herbal medicine)): ti, ab, kw
Mesh term #2: ((Omeprazole) OR (Proton Pump Inhibitor) OR (PPI) OR (Western medicine)): ti, ab, kw
Mesh term #3:((Gastric Ulcer) OR (GU) OR (Peptic Ulcer)): ti, ab, kw
Mesh term #4: ((clinical trials) OR (randomized controlled trials) OR (RCT))
#1 AND #2 AND #3 AND #4

### 3.3 Literature screening and data extraction

**Figure 1:**
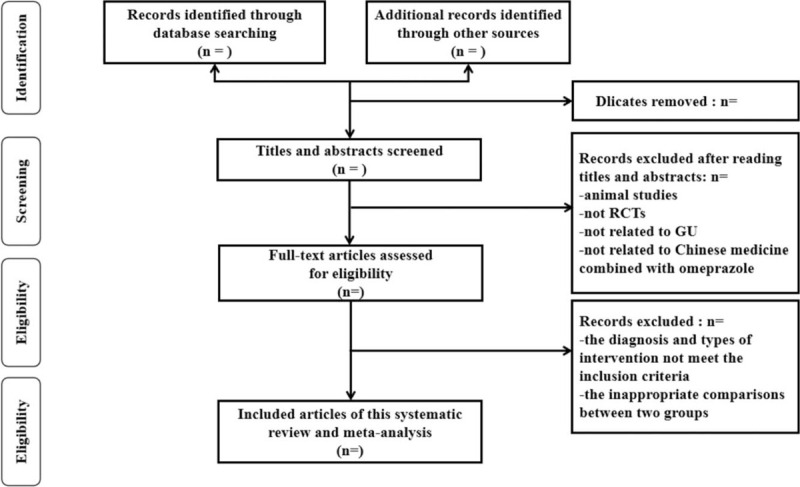
The PRISMA literature screening flow chart.

By reading the titles and abstracts of the articles one by one, preliminary screening is carried out, and the articles that obviously do not meet the inclusion criteria and those that have been submitted to one manuscript are eliminated. Read the full text according to the inclusion and exclusion criteria. For documents that provide incomplete information in the original text or have any ambiguities about their design or results, contact the author to obtain relevant information before making a decision. Read the full text that meets the inclusion criteria in the preliminary screening again, and read and analyze the original documents that may meet the inclusion criteria to determine whether they will be included in the final. Due to the heavy workload, errors are prone to occur in the screening and review process. To ensure quality, 2 people should individually screen the materials and evaluate the quality of the literature, and then crosscheck them. If there is a disagreement, both parties discuss or negotiate with a third party. The extracted literature information includes the first author and publication year, number of cases, intervention measures, course of treatment, outcome indicators, whether to use blinding, whether to use allocation concealment, whether to follow-up, whether to be lost to follow-up, etc. The selection process is illustrated in a PRISMA flow diagram (Fig. 1).

### 3.4 Risk of bias assessment

According to the RCT bias risk evaluation method recommended by Cochrane Handbook,^[9]^ the quality evaluation is carried out:

(1) random allocation;
(2) allocation hiding;
(3) blinding to the research objects and treatment plan implementers;
(4) evaluation of outcome indicators Blind method is used;
(5) report the result data completely;
(6) report the research results selectively;
(7) other sources of bias.

Two reviewers evaluated each article based on the above items, including 3 levels of “low risk of bias,” “unknown risk of bias,” and “high risk of bias.” For each included literature, 2 reviewers independently conduct methodological quality evaluation, and if there is a disagreement, they will discuss and resolve with the third person.^[10]^

### 3.5 Statistical analysis

#### 3.5.1 Measures of treatment effect

The RevMan 5.3 software provided by the Cochrane Collaboration was used for statistical analysis.^[11]^ Enumeration data uses risk ratio (RR) as the effect indicator, measurement data uses the mean difference (MD) as the effect indicator, and each effect size is given its point estimate and 95% confidence interval (CI).

#### 3.5.2 Assessment of heterogeneity

The heterogeneity test of the research results adopts the *χ*^2^ test. If the homogeneity is good (*P* > .1, *I*^2^ < 50%), the fixed-effects model is used for analysis; if the heterogeneity is large (*P* < .1, *I*^2^ > 50%), the heterogeneity is performed first sexual source analysis, and then subgroup analysis or sensitivity analysis based on possible heterogeneity factors, that is, random effects model and fixed-effects model are used. Whether there is a significant difference between the conclusions of the 2 different models for calculating the combined value of the effect. If the *Z* values are not much different and the *P* values are both meaningful, then the conclusions are robust and can eliminate heterogeneity. If the heterogeneity still exists, but the trials have clinical homogeneity, the random effects model is used to combine the effect size. However, if there is obvious clinical heterogeneity between the studies, they will not be merged, and only a descriptive analysis will be performed.

#### 3.5.3 Deal with the missing data

If there are missing data in the included studies, we will try to contact the corresponding authors of the relevant studies via email to obtain any necessary data. However, if the missing data cannot be obtained, the study will not be included in the statistical analysis.

#### 3.5.4 Publication bias

For outcome indicators with more included literature (≥10 articles), a funnel chart should be drawn to observe whether the points in the funnel chart are symmetrical and fall out to detect whether there is publication bias and small sample effect.^[12,13]^

#### 3.5.5 Subgroup analysis

If there is significant clinical and statistical heterogeneity (*P* < .1, *I*^2^ > 50%), subgroup analysis should be performed in order to further the source of heterogeneity. Group the influencing factors such as the type of intervention, age, race, etc, and observe the heterogeneous results.

#### 3.5.6 Sensitivity analysis

Carry out sensitivity analysis to test the reliability and stability of the system evaluation results, and look for the heterogeneity of causality. The method to solve this problem is to exclude the changes in the observation of heterogeneous results one by one from the included studies, to get rid of research bias or to remove the high risk of certain special studies.^[14]^

### 3.6 Ethics and dissemination

The studies we have included are all published documents and do not involve patients, so ethical approval is not required. The expected goal is to publish the results of this research in a peer-reviewed journal.

## 4 Discussion

In clinical application research, we found that the use of omeprazole alone has certain limitations. At present, Chinese medicine plays an indispensable role in treating GU and preventing recurrence due to its unique advantages. In terms of diagnosis and treatment, different physicians have different ideas of syndrome differentiation, different treatment methods, and a hundred schools of thought, which greatly enriched the theory of traditional Chinese medicine for the treatment of GU. The treatment of GU with traditional Chinese medicine is mainly to increase the protective factors of gastric mucosa and reduce the attack factors of gastric ulcer. It gives full play to the advantages of traditional Chinese medicine in multi-target and bidirectionality. Scholars have conducted research from different channels and links, and have achieved certain results at the molecular level.

This study compared the clinical efficacy of omeprazole alone and omeprazole combined with traditional Chinese medicine in the treatment of GU through a systematic review. However, the limitations of this systematic review indicate that this research focuses on the research of Chinese and English articles, and the included RCTs are still very few. Limited by the included research methodology, it needs to be confirmed by more multi-center, large sample, high-quality research.

## Author contributions

**Conceptualization:** Cheng Xie, Mingquan Wei.

**Data curation:** Langhui Liu.

**Formal analysis:** Suyou Zhu.

**Methodology:** Cheng Xie, Suyou Zhu, Mingquan Wei.

**Supervision:** Cheng Xie, Langhui Liu, Mingquan Wei.

**Writing – original draft:** Cheng Xie, Langhui Liu, Suyou Zhu.

**Writing – review & editing:** Cheng Xie, Mingquan Wei.

## References

[1] Sykes BW Sykes KM Hallowell GD. A comparison of two doses of omeprazole in the treatment of equine gastric ulcer syndrome: a blinded, randomised, clinical trial. Equine Vet J 2014;46:416–21. 24102898 10.1111/evj.12191

[2] Lau JY Sung J Hill C. Systematic review of the epidemiology of complicated peptic ulcer disease: incidence, recurrence, risk factors and mortality. Digestion 2011;84:102–13. 21494041 10.1159/000323958

[3] Liu J. Analyze the clinical effect of Chinese medicine Banxia Xiexin Decoction and western medicine in the treatment of patients with gastric ulcer due to weakness of the spleen and stomach. Digest World's Latest Med Information 2017;17:176–7.

[4] Fornai M Colucci R Antonioli L. Effects of esomeprazole on healing of nonsteroidal anti-inflammatory drug (NSAID)-induced gastric ulcers in the presence of a continued NSAID treatment: characterization of molecular mechanisms. Pharmacol Res 2011;63:59–67. 20969958 10.1016/j.phrs.2010.10.013

[5] Kangwan N Park JM Kim EH. Quality of healing of gastric ulcers: natural products beyond acid suppression. World J Gastrointest Pathophysiol 2014;5:40–7. 24891974 10.4291/wjgp.v5.i1.40 PMC4024519

[6] Ling Y. Clinical observation on treating gastric ulcer with TCM medicine and omeprazole. Clin Study Tradit Chin Med 2018;10:135–6.

[7] Zhou XJ Feng JY Sun BY. Clinical observation on treatment of peptic ulcer with combination of Chinese and Western medicine. J Aerosp Med 2012;23:1209–10.

[8] Shamseer L Moher D. Preferred reporting items for systematic review and meta-analysis protocols (PRISMA-P) 2015: elaboration and explanation. BMJ 2016;350:g7647 doi: 10.1136/bmj.g7647. 10.1136/bmj.g7647 25555855

[9] Cumpston M Li T Page MJ. Updated guidance for trusted systematic reviews: a new edition of the Cochrane Handbook for Systematic Reviews of Interventions. Cochrane Database Syst Rev 2019;10:D142. 10.1002/14651858.ED000142 PMC10284251 31643080

[10] Savović J Weeks L Sterne JA. Evaluation of the Cochrane Collaboration's tool for assessing the risk of bias in randomized trials: focus groups, online survey, proposed recommendations and their implementation. Syst Rev 2014;3:37. 24731537 10.1186/2046-4053-3-37 PMC4022341

[11] Wang C Weng H Li B. Application of RevMan 5.3 software for data transformation in etiological and prognostic meta-analysis. Chin J Evid Based Med 2017;17:852–6.

[12] Sutton AJ Duval SJ Tweedie RL. Empirical assessment of effect of publication bias on meta-analyses. BMJ 2000;320:1574–7. 10845965 10.1136/bmj.320.7249.1574 PMC27401

[13] Lin L Chu H. Quantifying publication bias in meta-analysis. Biometrics 2018;74:785–94. 29141096 10.1111/biom.12817 PMC5953768

[14] Yang W Zhang T Li Z. Combined analysis of endometrial thickness and pattern in predicting clinical outcomes of frozen embryo transfer cycles with morphological good-quality blastocyst: a retrospective cohort study. Medicine (Baltimore) 2018;97:e9577. 29480852 10.1097/MD.0000000000009577 PMC5943888

